# Supplementary material for: Chromothripsis during telomere crisis is independent of NHEJ, and consistent with a replicative origin
Source: Genome Res. 2019 May;29(5):737–49. doi: 10.1101/gr.240705.118 (PMC6499312; doi:10.1101/gr.240705.118)
Supplement: Supplemental Material [file supp_gr.240705.118_Supplemental_file_1.zip › contigs/annotated_contigs/DB38/contig.2.DB38_length_771_mean_cov_40.6018158236.docx]

**DB38_length_771_mean_cov_40.6018158236**

```
TCCTTCCTTCCTTCCTTTCTTCCTTTCTTTCTTTCTTTCTTTCTTTCTTTCTTTCTTTCTTTCTTTC|TTTCTTTCTTTCT|TTCTTTT
 >chr6:133113364-133113444 - E=3e-36 p=0e+00                        >chrUn_gl000220:102
GTTTCTTTCTTTCTTGCTTTCTTGCTTGCTTGCTTTCTTGCTTTCTTGCTTGCTTGCTTTCGTGCTTTCTTGCTTTCTTTTCTTTCTTT
897-103592 + E=0e+00
CTTTTCTTTTTCTTTCTTTCTTTCTTTCTTGCTTTCTTTTCTTTCATTCATTCCTTCTTTCTTTTCTTTCTTTCTTCCTTCCTTCCTTC

CTTCCTTCCTTCCTTTCTTTCTTTCTTTCTGTTTCGTCCTTTTGAGACAGAGTTTCACTCTTGTTTCCACGGCTAGAGTGCAATGGCGC

GGTCTTGGCTCACCGCACCTTCCGCCTCCCGGGTTCGAGCGCTTCTCCTGCCTCAGCCTCCCGATTAGCGGGGATTACAGGGAGGCACC

CCCACGCCTGGCTTGGCTGATGTTTGTGTTTTTAGTAGGCACGCCGTGTCTCTCCATGTTGCTCAGGCTGGTCTCCAACTCCCGACCTC

CTGTGATGCGCCCACCTCGGCCTCTCGAAGTGCTGGGATGACGGGCGTGAGCCACCGTGCCCGGCCTGTTGACTCATTTCGCTTTTTTA

TTTCTTTCGTTTCCACGCGTTTACTTATATGTATTAATGTAAACGTTTCTGTACGCTTATATGCAAACAACGACAACGTGTATCTCTGC

ATTGAATACTCTTGCGTATGGTAAATACGTATCGGTTGTA|CGGGAATTAACCTCTCTATGA
```
